# Current News

**Published:** 1902-12

**Authors:** 


### Current News.

### INSTITUTE OF DENTAL PEDAGOGICS: TENTH ANNIVERSARY.

THE tenth annual meeting of the Institute of Dental Pedagogics will be held in Chicago, December 29, 30, and 31, 1902, at the Palmer House. All who are interested in Dental Education are cordially invited to attend. The following programme has been arranged:

PAPERS.

"Teaching Operative Procedure," Dr. C. N. Johnson.

"Teaching General Anatomy to Dental Students," Dr. Borland.

"Teaching Electricity and its Dental Uses," Dr. W. A. Price.

"Teaching Embryology," Dr. I. N. Broomell.

"Teaching Applied Physics," Dr. G. V. Black.

"Physical Diagnosis."

A Symposium on the "Management of the Teaching of Demonstrators in the Infirmary," by four professors.

Report of Committee on "Four-year Curriculum."

Report of Committee on "Nomenclature."

All new teaching appliances must be submitted to Dr. Whitslar, of Cleveland, or to Dr. Patterson, of Kansas City.

Hart J. Goslee,
*President.*
W. Earl Willmot,
*Chairman Executive Board.*
H. B. Tileston,
*Secretary and Treasurer.*

---

### PENNSYLVANIA ASSOCIATION OF DENTAL SURGEONS.

The Pennsylvania Association of Dental Surgeons held its fifty-sixth annual meeting on the evening of October 14, 1902, at the Continental Hotel, Philadelphia. The following officers were elected to serve during the ensuing year:

President, Dr. Wilbur F. Litch; Vice-President, Dr. Geo. W. Cupit; Secretary, Dr. J. Clarence Salvas; Treasurer and Librarian, Dr. Wm. H. Trueman.

During the past year the following papers were read and discussed before the Society: "Combination Fillings," by Dr. Joseph Head; "Obtundents," by Dr. Chas. S. Moore; "The Difference in Method of High- and Low-Fusing Porcelain for filling Teeth," by Dr. W. A. Capon; "A Practical View of the Plastics," by Dr. J. Clarence Salvas; "Alveolar Abscess: Its Complications and Treatment," by Dr. J. F. Wessels; "The Danger of Infection of the Eye of the Dentist while operating," by Wendal Reber, M.D.; "Calcification of the Dentine and Enamel and its Relation to Hypersensitiveness of these Tissues," by Dr. I. N. Broomell; "Adenoids and their Relation to Oral Deformity," by Dr. M. I. Schamberg; "A Sketch of Edward Hudson," by Dr. Wm. H. Trueman; "General and Local Anæsthesia, with Special Reference to its Application in Operations within the Mouth," by Dr. E. Quin Thornton, M.D.

J. Clarence Salvas,
*Secretary.*

